# Effect of Oxygen-Reducing Atmospheres on the Safety of Packaged Shelled Brazil Nuts during Storage

**DOI:** 10.1155/2011/813591

**Published:** 2011-07-07

**Authors:** Vildes Maria Scussel, Barbara Nantua Giordano, Vanessa Simao, Daniel Manfio, Simone Galvao, Manuel Nazaré Ferreira Rodrigues

**Affiliations:** Laboratory of Mycotoxicology and Food Contaminants, Department of Food Science and Technology, Center of Agricultural Sciences, Federal University of Santa Catarina, P.O. Box 476, Florianopolis, SC, Brazil

## Abstract

This work reports the application of oxygen-(O_2_-) reducing atmosphere methods on stored shelled Brazil nut (*Bertholletia excelsa* H.B.K.) packs aiming to evaluate the degree of aflatoxin degradation, nuts lipid oxidative stability, fungi control, and hygienic conditions improvement. The methods applied were (a) ozone: O_3_, (b) carbon dioxide: CO_2_, and (c) O_2_ absorber pads with and without vacuum. From all modified atmospheres evaluated, the best performance was obtained with O_3_, either with or without vacuum. It was the only nut treatment that was able to degrade aflatoxins. None of the spiked (AFLs: 15 *μ*g·kg^−1^) nut samples O_3_- treated had aflatoxins detected up to the LC-MS/MS method LOQ (0.36 *μ*g·kg^−1^ for total AFLs), thus producing safer nuts. Also it kept the fatty acid oxidation indicator—malondialdehyde stable and improved the sensory attributes for consumer acceptance. In addition, the destruction of fungi and yeast was observed since the O_3_ application (from 1.8 × 10^4^ cfu/g to NG = no growth). All other treatments stabilized and/or inhibited microorganisms' growth only. By adding CO_2_ gas also played an important role in the nut quality. Regarding cost, gaseous O_3_ showed to be of low cost for application in the nut packs.

## 1. Introduction

In nature, Brazil nuts (*Bertholletia excelsa* H.B.K.) that grow in the Amazon forest may get contaminated by fungi and aflatoxins [1–3], as other tree nuts. The aflatoxigenic *Aspergillus* species that have been isolated from Brazil nuts are *A. flavus, A. parasiticus,* and *A. nomius* [4–7]. Their growth is directly related to the climate conditions of that region and to the conditions during their storage, transport, and commercialization, if there is no control of moisture content (m.c.) and temperature. That can also occur if nuts are packaged in a microclimate rich in oxygen (O_2_) and m.c. enough to allow microorganisms to grow [1, 8]. 

Studies have reported the use of modified atmospheres (MA) in food storage, extensive to packaging, to reduce O_2_ concentration by adding gases such as nitrogen, carbon dioxide (CO_2_), and ozone (O_3_) which lead to microorganisms (fungi, yeast, and bacteria) inhibition, maintenance of lipid stability, and reduction of grains/nuts/vegetable respiration [9–14]. Vacuum also is an alternative for O_2_ reduction and in recent years the addition of O_2_ absorber pads (which contains a mixture of iron salts) have been the newest alternative in packaged food [15–17]. Studies have reported O_3_ and CO_2_ effect on controlling microorganism growth in several agricultural commodities [13, 18–21]. CO_2_ is a promising and efficient inactivating microorganisms' gas for application on nonthermal sterilization process [22, 23]. Maeba et al. (1988) reported the destruction and detoxification of AFB_1_ and AFG_1_ in agricultural products treated with 1.1 ppm of O_3_ during 5 minutes [24]. Aflatoxin degradation in different food products, either fresh or processed at different O_3_ concentrations, has been reported by some authors [13, 25–28]. An advantage of gaseous O_3_, apart from being a powerful disinfectant, oxidant, and aflatoxins degrader, is that it decomposes quite fast into O_2_ and does not have toxic effect [29–31]. 

**Figure 1:**
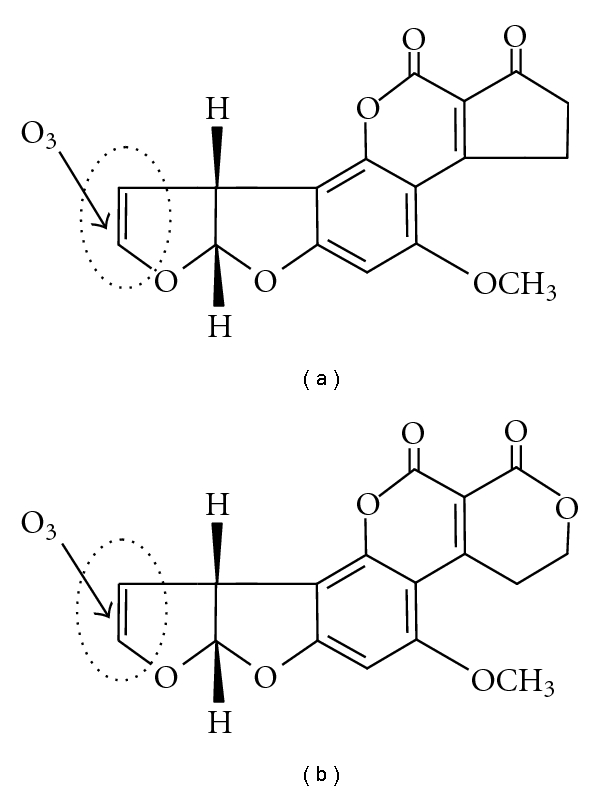
Chemical structures of the two more toxic aflatoxins, (a) AFB_1_ and (b) AFG_1_, with the sites of ozone oxidative attack (8,9 double bond of final furan rings in both structures).

The degradation of mycotoxins by O_3_ is found to follow a pseudo-first-order rate, as long as a continuous supply of O_3_ is maintained [8]. For aflatoxins, the higher degradation rate for AFB_1_ and AFG_1_ was attributed to the presence of an 8,9 double bond forming a vinyl ether at the terminal furan ring (Figure 1), which is not present in AFB_2_ and AFG_2_. These latter forms require longer exposure due to a possible second mechanism, when the lactone ring is opened during O_3_ exposure [29].

High m.c., relative humidity, temperature, and environment rich in O_2_ are the main factors for tree nuts to get aflatoxin contaminated and so infected by fungi. During storage and commercialization dry shelled Brazil nut packs need to maintain their safety and quality. Considering that MA in storage (macroenvironment) and packaging (microenvironment) can prolong food shelf life by reducing O_2_ concentration, this work reports the application of O_2_-reducing atmosphere methods (vacuum, CO_2_, O_3_, and O_2_ absorber) on fungi reduction, aflatoxin degradation, and lipid stability during storage of snack packs of shelled Brazil nuts.

## 2. Material and Methods

### 2.1. Sample

Shelled dry (processed) Brazil nuts (25 kg). They were provided by the Renmero Factory from Cameta city, Para State, Northern Brazil. The nuts type and conditions were as follows: (a) medium size (40–50 mm length [32]); (b) initial m.c. and total fungi load of 6.5% and 1.83 × 10^4^ cfu·g^−1^, respectively; (c) no aflatoxin contaminated (method LOQ: 0.36 *μ*g·kg^−1^); (d) absence of coliforms, *Salmonella* and *Staphylococcus*. That batch was utilized for the aflatoxin spiking experiment. A special nut batch (10 kg) *naturally* aflatoxin contaminated (10.61 *μ*g·kg^−1^) was used for further aflatoxin O_3_ degradation comparison. Its m.c. and total fungi load were 7.2% and 3.7 × 10^4^ cfu·g^−1^, respectively. Nuts 260 g portions were prepared for the experiments.

### 2.2. Application of O_2_-Reducing Atmospheres

Shelled Brazil nuts were divided into two groups. (a) *Group I*: *control:* nuts packed (a.1) loose: only air inside and (a.2) under vacuum. (b) *Group II*: *aflatoxin spiked* (15 *μ*g·kg^−1^): nuts were divided into subgroups and packed (b.1) loose: only air inside; (b.2) vacuum; (b.3) O_3_ treated (packed with and without vacuum); (b.4) CO_2_ gas added into packs; (b.5) O_2_ absorber pads (packed with and without vacuum). The series O_3_ (concentration: 10.0 mg/L, 90 min—[21]) was applied on the nuts separately and then aseptically packaged. The O_3_ concentration checking was performed by the iodine metric test [33]. (c) *Group II*: *naturally aflatoxin contaminated* (10.6 *μ*g·kg^−1^): nuts O_3_ treated were packed with and without vacuum. The O_3_, CO_2_ gas, and O_2_ absorber pad application was carried out utilizing an O_3_ generator (MZ01, MegaZon, Pondicherry, India), a CO_2_ cilinder (White Martins, Jundiaí, SP, Brazil), and O_2_ pad (Ageless, New York, USA), followed by sealing and/or vacuum + sealing by means of a vacuum machine with heat sealer (Sunnyvale, CA, USA). The snack packs (O_2_ and UV barrier polypropylene film, 20 × 25 cm length × width) filled with 260 g nut portions each and treated, were stored in an BOD incubator (Dist, Florianopolis, SC, Brazil.) at 27°C during two months.

**Sample Collection for Analysis**

**Figure 2:**
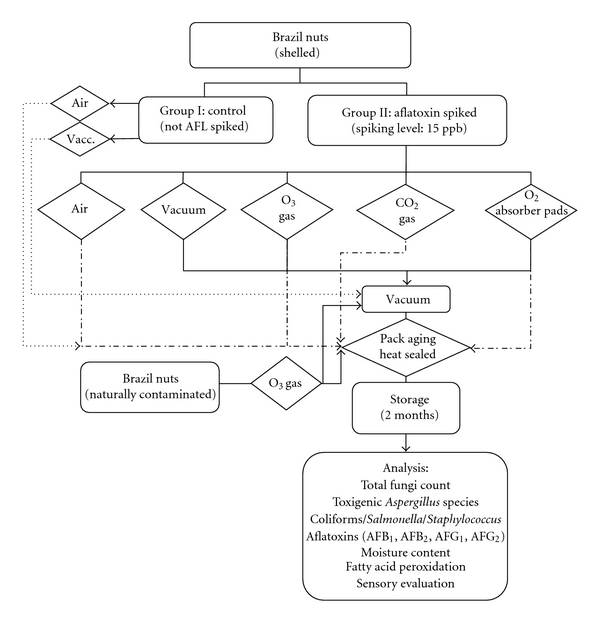
Chart flow of the oxygen-reducing atmospheres application on shelled Brazil nuts packs during storage.

Individual packs of shelled Brazil nuts were collected at Day one (after each treatment) and every 30 days (triplicate *n* = 3). See flowchart of the whole experiment in Figure 2.

### 2.3. Shelled Brazil Nut Analysis

(a) *Microbiological methods*: for total fungi count the method was of Pitt and Hocking (1997) [33]; the presence of *Aspergillus* species was checked utilizing the Aspergillus flavus and parasiticus agar (Fluka, St. Gallen, Switzerland) by Pitt et al. (1983) [34]; the identification of fungi in genus and species was carried out according to the keys of Samsom et al. (2004) [35] and *Salmonella* spp.*, Staphylococcus* spp., and coliforms (45°C) were checked by APHA (1997) [36]. (b) Aflatoxin *determination*: was carried out by LC tandem mass spectrometry [37]. Briefly, aflatoxins (Sigma, Zwijndrecht, The Netherlands) were extracted from ground Brazil nuts with acetonitrile : water (HPLC grade, Carlo Erba, Milan, Italy and MilliQ, Millipore, Bedford, MA, USA, resp.) at 80 : 20 v/v, mixed, filtered, and injected into an Waters Alliance 2695 separation module with a 20 *μ*L injection loop (Waters, Milford, USA) and a C_18_ column 150 × 3.2 mm, 5 *μ*m (Alltech, Breda, The Netherlands) at 30°C. Separation was performed utilizing methanol (Carlo Erba) : water (both with 25 mM of ammonium acetate, J. T. Baker, Phillipsburg, NJ, USA) as mobile phase at 1 mL·min^−1^ of flow rate. The LC system was coupled to a Quatro Ultima triple quadrupole mass spectrometer (Micromass, Manchester, UK) and toxins were detected and quantified by using atmospheric pressure chemical ionization in the positive mode [M+H]^+^. For details on equipment settings, please refer to the method. (c) *Moisture content* was determined by gravimetry [38]. (d) *Fatty acid oxidation* was determined by the TBA method of Genot (1996). Extraction with 5% TCA (J. T. Baker) containing freshly prepared BHT in ethanol (J. T. Baker and Carlo Erba, resp.). After filtration, extract was mixed with TBA (J. T. Baker,) and immersed in a 70°C water bath (Dubnoff Q226D, Quimis, Diadema, Brazil) for 30 min, cooled in ice and the absorbance of the reacted solutions read at 532 nm (spectrophotometer E005, Hitashi, Tokyo, Japan) against a blank containing TCA and TBA reagents. The results expressed as mg of malondialdehyde (MDA) equivalents per kilograms nut sample (LOQ: 0.37 mg·kg^−1^) [39]. (e) *Sensory evaluation* was based on the descriptive quantitative analysis [40]. Eighteen trained panelists during four sessions (*n* = 4) described impressions perceived by the hedonic scale of 5 points (1: dislike very much, 2: dislike, 3: neither like nor dislike, 4: like and 5: like very much). Sensory attributes evaluated: nut appearance (AP), color (CO), firmness (FI), resistance to slicing (SR), rancid (RA), and strange (OD) odors. 

### 2.4. Statistical Analysis

The results were expressed as the mean values and standard errors. Statistical analysis was performed by analysis of variance (ANOVA) and included the Tukey's test to evaluate significant differences among the means (*P* < 0.05). Figure 2 shows the flowchart on the whole study.

## 3. Results and Discussion

All the MA-treated shelled Brazil nut packs presented better quality and safety than the MA-untreated nuts (Group I: air) throughout the whole storage period. It was observed different degrees of fungi reduction and in some groups, aflatoxin degradation too.Table 1 shows the safety (fungi load; aflatoxins) and quality (m.c.; fatty acid oxidation; sensory evaluation) data obtained from the different MA-treated nut Groups (I, II, and III).

### 3.1. MA Effects on Shelled Brazil Nuts Microbiological Content and M.C

As expected, inhibition of microoganisms growth was registered throughout the experiment despite the MA applied.

**(a) Total Fungi Load and Aflatoxigenic Strains**

A substantial fungi reduction was observed, both with O_2_ absorber and O_3_ packaged under vacuum, as well as with nuts O_3_ loose pack ones. CO_2_ also played an important role in the microorganism reduction in the current experiment reducing from 1.8 × 10^4^ cfu·g^−1^ to NG (no grow). Applying vacuum improved quality and safety regarding fungi further. Although, it was observed a reduction on their growth in the MA-treated Groups; in the untreated nuts (Group I) was possible to isolate and identify them. Their main genera and species were *Acremonium sp; A. ochraceus*; *Cladosporium sp.; P. corylophilum Rhizopus sp.* followed by *A niger; A. parasiticus; A. versicolor; P. crustosum*. With regards to m.c., nuts presented different degree of reduction after being MA-treated as follows: O_3_ + vac > vac > O_2_  abs + vac > O_3_ > CO_2_. That was especially true for vacuum treated packs, which led to a synergistic effect (low m.c. + lack of O_2_) on controlling fungi growth. Regarding the O_3_ treated nuts, the reduction of m.c. was due to the fact that during O_3_ application occurred an exposure of nuts to 90 minutes with O_3_ stream that can take moist from nut surface apart from its known reaction with atmospheric water, decreasing the microenvironment relative humidity [21]. In fact, the lowest total fungi count, that is, no growth was detected in the packs that nuts were submitted to O_3_ with or without vacuum application (m.c. reduction: −1.8 and −3.5%, resp.), suggesting that apart from the fungi destruction by the O_3_, the reduction of m.c. powered fungi reduction. These data were corroborated by some authors that reported m.c. reduction in different foods including in-shell Brazil nuts O_3_ treated [1–3, 21].

**(b) Hygienic Bacterial Indicators**

Similar to what was observed for fungi and yeast, all gases and O_2_ absorbers as well as vacuum did not allow *Salmonella*, *Staphylococcus,* or coliform to grow on the nuts showing the safe power of the treatments for microbial population control. It is important to emphasize that the potent disinfectant characteristics of O_3_ has been recognized by the Food and Agriculture Organization [41] and Food and Drug Administration [42].

### 3.2. MA Effects on Shelled Brazil Nuts Aflatoxin Degradation

**Figure 3:**
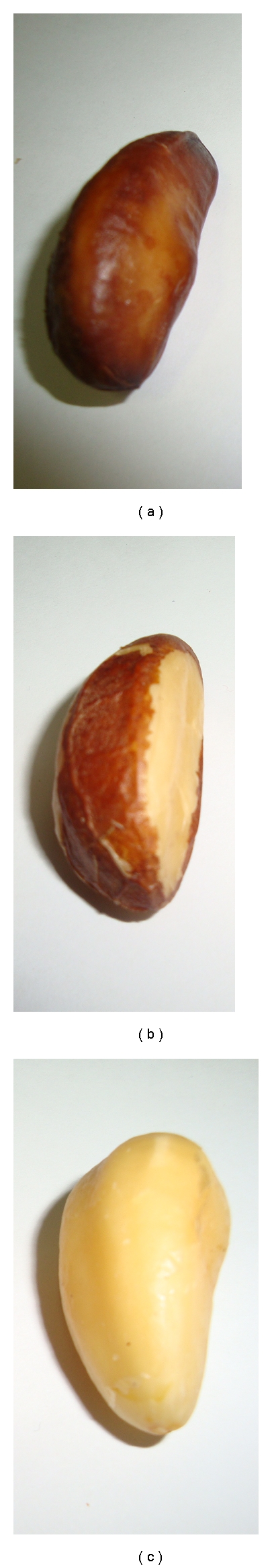
Shelled dry Brazil nuts (a) totally protected by the *testae,* (b) with some remained *testae*, and (c) totally cleaned for packaging, showing its sealed nut surface.

It was possible to observe in the aflatoxin spiked nut samples O_3_ treated (Groups II: O_3_ and O_3_+vac) that the gas was able to degrade them as none, during the storage period, were detected (LC-MS/MS method LOQ: 0.36 *μ*g·kg^−1^). That was different for the other O_2_ reducing atmospheres (CO_2_ and O_2_ absorber pads with/without vacuum). They were able only to stabilize/reduce the microorganisms growth keeping nuts safe but with aflatoxins. In that sense the packs with O_3_ and vacuum applied bring an alternative for aflatoxin degradation and also m.c. reduction, a factor that is directly related to fungi proliferation and development of possible aflatoxigenic strains.Nuts O_3_ treated utilized in the study showed to be able for consumption, as no aflatoxin was detected in none of them. *Brazil nuts naturally contaminated by O_3_*: to make sure O_3_ would degrade aflatoxins not only in spiked nuts (i.e., toxins just applied and dried onto nut surfaces), we carried out also an experiment utilizing the special batch of nuts naturally aflatoxin-contaminated (packs with nuts O_3_ and O_3_ + vacuum treated). Similarly to the nuts spiked, no aflatoxin was detected after O_3_ application neither fungi. That was probably due to the fact that Brazil nut has the advantage of its contamination/fungi proliferation to occur mostly on the nut surface/external layer as its structure is completely sealed (Figure 3). In addition, the *testae* (a pellicle that surrounds the edible part) of the Brazil nut, which is rich in Selenium (antioxidant), can act as a protector [2]. Thus reducing the possibility of easy access by the fungi spores to the nut core, as it occurs in peanuts (loose *testae*) or shelter fungi spores in-between shell and edible part of pistachios (in-shell), making gaseous O_3_ application and action more effective. Currently, there is no available technology to completely eliminate the mycotoxin contamination of food and feed chain. Most of the current strategies for mycotoxin reduction are based on prevention, either pre- or postharvest and detoxification, which are not always effective. From the available tools to ensure food safety, O_3_ application may be one of the most promising methods that come to meet the grain producers and food industries needs [43–45].

### 3.3. O_2_-Reducing Atmosphere Effect on Nuts Quality

As far as quality is concerned, the parameters evaluated were the fatty acid oxidation, m.c., and sensorial evaluation which give information on lipid rancidity development, crunchiness texture alterations, and aroma/colour/odor/texture modifications. (a) *Fatty acid peroxidation:* regarding MDA formation during the nuts storage period and MA applied, no significant changes occurred despite the MA applied except for the O_2_ absorber at the end of the storage period. In contrary, the samples packed loose in air (either Control and spiked) had an increase of MDA from 7.24 to 9.98 mg·kg^−1^. With regards to CO_2_ and O_3_, with and without vacuum, effect on shelled Brazil nuts lipids, it was observed that the values of MDA lowered and kept constant throughout the whole period of storage (Table 1). The same occurred when Gamli and Hayoğlu (2007) studied vacuum packaged pistachio [46]. The authors observed no significant difference on the MDA values during the storage period and reported that those results could be attributed to the higher amount of the fatty acid oleic acid (monounsaturated) and less linoleic acid (poliunsaturated) content in that nut. These results can be attributed to the reduction/control in the oxidation rate speed, both, by air withdraw (vacuum) and O_3_ treatment (waste from O_2_ removal). Similar results occurred in peppers and pistachio after the application of O_3_ and vacuum packaging, that is, the effect on lipid oxidation was not apparent, thus could not alter the sensory characteristics [13] which was corroborated with the current data of the Brazil nut experiment. It is different when Rudolph et al. (1992) evaluated the oxidative stability of pecan oil and observed that changes in colour (O_2_ effect on carotenoids) followed by a rapid increase of rancidity products (O_2_ effect on fatty acids) [47]. However in the case of oil, lipids (fatty acids) present much more intense exposure to air O_2_ than when it is protected in the liposomes inside the nut cells (or just the nut damaged surface exposes their lipid content from broken cells/lysosomes. As for Brazil nuts where their oil (lipids) is protected inside the undamaged nut cells or slightly damaged during the industrial nut cracking procedure. (b) *Sensory Evaluation:* the scores for the sensory attributes tested (nut appearance, strange odor, rancid odor, slicing resistance, and firmness) are shown in Table 1. The O_2_ absorber pads applied led to some slight variation with regards to the visual appearance (color) of nuts probably due to its reducing effectiveness in the nuts located away from the pad site, sitting for long time (60 days) during storage. The sensory analysis of the Brazil nuts treated with O_3_ and vacuum-packed did not present significant changes among the panelists (*P* < 0.05). All scores for the O_3_-treated nuts during storage period were between 4 (like) and 5 (like very much). It was verified also that O_3_ leaves no residual odor. In fact O_3_ with vacuum and vacuum only received the best scores showing that vacuum is still the best choice when preserving sensory characteristics is concerned. Similar thing occurred when Inan et al. (2006) worked with red pepper ozonation [27]. They did not register significant sensory changes after the O_3_ application as the peppers were still quite palatable. When Akbas and Ozdemir (2006) studied the quality of pistachio, no significant changes also were observed between sweetness, rancidity, overall appearance, and taste, compared to control samples (no O_3_) indicating the efficacy of that gas application [13]. Other authors also have reported the efficiency of the O_3_ and its low interference in the sensory attributes of quality in several products such as vegetables, fish, birds carcasses, and their byproducts [44, 48, 49]. In a work carried by Dull and Kays (1988) with pecans, the author reported a slight better sensory quality on the vacuum-treated packaging nuts after 6 months of storage at 24°C. Other potential uses of O_3_ in the food industry include reduction of undesirable volatile metabolites, such as off-flavours or contaminants by their removal during O_3_ stream application [50]. Considering that MDA is volatile, all experiments that had vacuum applied and/or gaseous O_3_ stream exposure, had lower MDA just after application (day one), thus giving the idea of fast oxidation reduction which was expected. The nuts final storage MDA measure should be taken as the indicator of the degree of oxidation together with the sensory evaluation. (c) *Moisture content:* as expected, nuts presented m.c. reduction after the MA treatments. That was especially true for vacuum-treated packs which kept nuts cruncher throughout the whole storage period. That effect was enhanced by the O_3_ application which allowed to reduce possible off-odours.

## 4. General Discussion

All O_2_-reducing atmospheres treated Brazil nut packs presented better nut quality after the period of study. However, the best performance, regarding safety was obtained either with or without vacuum. It was the only nut treatment that was able to degrade aflatoxins. It also led to fungi/yeast destruction, was able to eliminated off-flavours, reduced m.c., and maintained fatty acid oxidative process stable thus leading to safer, cruncher, and of better quality nuts. Next comes vacuum that kept sensory attributes of consumer acceptance, kept controlled lipid oxidation, and microorganisms. All other treatments stabilized and/or inhibited microorganisms' growth only, which also is important regarding safety, microorganisms-wise, and quality nuts. 

 Considering that Brazil nuts can be suitable to aflatoxin contamination, are good fungi substrate, and are rich in oil, as other tree nuts, the best method that could control those parameters and improve consumer acceptance for best and stable dry nut product packs is O_3_ with vacuum. Aflatoxin that may still remain in the Brazil nuts at the packaging can be destroyed at that stage. O_3_ will be useful for those hard packages (tubs) sold in small portion, that are commonly commercialized in Brazil too—providing a safer product for the national consumers. Regarding the costs and environment impact, O_3_ equipments are of low cost and environment friendly, it is fast converted into O_2_. 

Our modern days have been emphasizing the importance of additives reduction in food. The use of O_3_ features as an important technology for food storage and industry as it leaves no residue. Its use has been approved in countries around the world and supported by several international food and health agencies inclusive for use in organically labelled agricultural products, inclusive in medicine. Currently, there is no available technology to completely eliminate the mycotoxins contamination of food and feed chain. Most of the current strategies for mycotoxin reduction are based on prevention, either pre- or postharvest. From the available tools to ensure food safety, O_3_ application is one of the most promising methods that come to meet the needs.

## Figures and Tables

**Table 1:** Effect of O_2_ reducing atmospheres on aflatoxins, lipids, microorganisms and consumers acceptance of packaged shelled Brazil nuts during storage.

Storage		Total fungi count*(cfu·g^−1^)	*Aspergillus* toxigenic strains	m.c.^a^		Aflatoxins^b^ (*μ*g·kg^−1^)	Lipid stability (mg·kg^−1^)^c^	Brazil nuts sensory attributes (scores)^d^					
Atmosphere	Day			(%)	(diff.%)^e^			AP	CO	FI	RA	SR	OD
GROUP I—Control^f^													

*Air*													

	Initial	1.83 × 10^4^	(+) *A. flavus; A. parasiticus * ^h^	6.5		<0.36	7.24 ± 0.9	4	4	4	4	4	4
	Final	2.69 × 10^7^	(+) *A. flavus; A. parasiticus *	8.1	(+0.6%)	<1.89	9.98 ± 1.6	2	3	3	2	3	2

*Vacuum*													

	Initial	1.83 × 10^4^	(+) *A. flavus; A. parasiticus *	4.2		<0.36	7.33 ± 0.4	4	4	4	4	4	4
	Final	0.70 × 10	(+) *A. flavus; A. parasiticus *	4.2	(no diff.)	<0.36	7.24 ± 1.1	4	4	4	4	4	4

GROUP II^i^													

*Air*													

	1	1.83 × 10^4^	(+) *A. flavus; A. parasiticus *	6.5		15.00	7.33 ± 0.1	4	4	4	4	4	4
	30	2.96 × 10^5^	(+) *A. flavus; A. parasiticus *	7.1		15.81	8.25 ± 0.2	4	3	4	3	3	3
	60	6.30 × 10^6^	(+) *A. flavus; A. parasiticus *	7.1	(+0.6%)	16.85	9.24 ± 0.7	3	3	3	1	3	2

*Vacuum*													

	1	1.83 × 10^4^	(+) *A. flavus; A. parasiticus *	4.2		15.00	6.85 ± 0.5	4	4	4	4	4	4
	30	0.56 × 10	NG^j^	4.2		14.88	7.24 ± 0.8	4	4	4	4	4	4
	60	0.10 × 10	NG	4.2	(−2.3%)	14.95	7.24 ± 0.5	4	5	5	4	4	4

*Ozone*													

	1	NG	NG	5.0		<0.36	7.25 ± 1.2	4	4	4	4	4	4
	30	NG	NG	4.9		<0.36	7.24 ± 0.6	4	4	4	4	4	4
	60	NG	NG	4.7	(−1.8%)	<0.36	7.84 ± 0.6	4	5	4	4	4	4

*Ozone* + *vacuum*													

	1	NG	NG	3.1		<0.36	6.25 ± 0.2	4	4	4	4	4	4
	30	NG	NG	3.3		<0.36	7.04 ± 0.7	4	4	4	4	4	4
	60	NG	NG	3.0	(−3.5%)	<0.36	7.25 ± 0.5	4	4	5	4	4	4
*Carbon* *dioxide*													

	1	1.83 × 10^4^	(+) *A. flavus; A. parasiticus *	6.5		15.00	7.25 ± 0.2	4	4	4	4	4	4
	30	NG	NG	7.0		14.90	7.24 ± 1.1	4	4	4	4	4	4
	60	NG	NG	7.0	(+0.5%)	14.92	7.90 ± 0.6	3	4	4	4	4	3

*Oxygen* *absorber* *pad*													

	1	1.83 × 10^4^	(+) *A. flavus; A. parasiticus *	6.5		15.00	7.25 ± 2.2	4	4	4	4	4	4
	30	2.6 × 10	NG	6.5		14.90	7.90 ± 1.7	4	4	4	4	4	4
	60	NG	NG	6.5	(no diff.)	15.00	8.20 ± 0.6	4	3	4	4	4	3

*Oxygen* *absorber * *pad + vacuum *													

	1	1.8 × 10^4^	(+) *A. flavus; A. parasiticus *	4.0		15.00	7.00 ± 2.2	4	4	4	4	4	4
	30	NG	NG	3.9		15.01	7.24 ± 1.5	4	4	4	4	4	4
	60	NG	NG	4.3	(−2.2%)	14.99	7.24 ± 1.5	4	3	5	4	4	3

GROUP III^k^													

*Ozone*													

	1	NG	NG	5.6		<0.36	7.56 ± 0.9	4	4	4	4	4	4
	30	NG	NG	5.4		<0.36	7.94 ± 0.2	4	4	4	4	4	4
	60	NG	NG	5.2	(−1.6%)	<0.36	7.99 ± 0.5	4	4	3	4	4	4

*Ozone* + *vacuum*													

	1	NG	NG	4.0		<0.36	7.95 ± 0.4	4	4	4	4	4	4
	30	NG	NG	3.9		<0.36	7.94 ± 1.2	4	4	4	4	4	4
	60	NG	NG	3.7	(−3.2%)	<0.36	8.54 ± 0.6	4	3	4	4	4	4

^a^m.c.: moisture content; ^b^aflatoxin total: AFB_1_+AFB_2_+AFG_1_+AFG_2_ (method LOQ: 0.350 *μ*g/kg); ^c^in malondialdehyde; ^d^values as mean scores of 18 individual panellists [AP: nut appearance; CO: color; FI: firmness; RA: rancid odour; SR: slicing resistant; OD: strange odor (5: like very much, 4: like, 3: neither like nor dislike, 2: dislike and 1: dislike very much)]; ^e^diff: m.c. difference (+) increased or (−) reduction; ^f^no aflatoxin spiked (nuts total AFL < method LOQ = 0.36 *μ*g·kg^−1^); ^h^toxigenic *Aspergillus* strains isolated in AFPA media; ^i^15 *μ*g·kg^−1^ AFLs spiked and 6.5% m.c.; ^j^NG: no growth; ^k^Brazil nuts naturally aflatoxin contaminated = 10.61 *μ*g·kg^−1^ and 7.2% m.c. *The genera and species more often isolated from the Control Brazil nuts were *Acremonium sp; A. ochraceus*; *A. nomius; Cladosporium sp.; P. corylophilum, and Rhizopus sp.* followed by *A. niger; A. parasiticus; A. versicolor, and P. crustosum*.
